# The use of Endo barrier for prevention of COVID‐19 infection enables upper gastrointestinal endoscopy with high patient satisfaction in private clinic

**DOI:** 10.1002/deo2.204

**Published:** 2023-01-02

**Authors:** Atsushi Imagawa, Hideki Kobara, Noriko Nishiyama, Tsutomu Masaki

**Affiliations:** ^1^ Department of Gastroenterology Imagawa Medical Clinic Kagawa Japan; ^2^ Departments of Gastroenterology and Neurology Faculty of Medicine Kagawa University Kagawa Japan

**Keywords:** COVID‐19, Endo barrier, esophagogastroduodenoscopy, medical staff, patient satisfaction

## Abstract

**Objectives:**

In the midst of the coronavirus disease 2019 (COVID‐19) infection pandemic, practitioners who perform endoscopic examinations need to prevent infections through procedures, along with routine medical care. By using continuous suction, Endo barrier is thought to be effective in preventing the spread of severe acute respiratory syndrome coronavirus 2 (SARS‐CoV‐2) droplets and aerosols. The study aimed to evaluate patient discomfort and satisfaction with the use of the Endo barrier during esophagogastroduodenoscopy (EGD). The study evaluated the system's effectiveness as well as the system's preparation time and the amount of burden on the medical staff.

**Methods:**

EGD was performed on 788 consecutive cases using the Endo barrier. A questionnaire was used to survey patients after the procedure on four points: discomfort (feeling of pressure, breathlessness) and good points (feeling of relief and satisfaction) using a visual analog scale. In addition, patients were divided into two groups according to sedation status: with sedation (69.7%) and without sedation (30.3%), and their scores were compared. Additionally, the preparation time of the Endo barrier was measured.

**Results:**

Patient discomfort was reported as minimal, resulting in a high level of satisfaction using this system. Although the overall results were better in the sedation group, the overall evaluation of the non‐sedated group was also favorable. Furthermore, preparation time (30 s) was less burdensome for medical staff.

**Conclusion:**

The Endo barrier is an easy‐to‐implement tool to prevent COVID‐19 infection in private clinics, and both patients and staff were highly satisfied with the device with or without the use of sedation during EGD.

## INTRODUCTION

As of September 2022, more than 612 million confirmed cases and 6.5 million deaths worldwide have been reported as a result of coronavirus disease 2019 (COVID‐19) infections.^1^ Private practitioners are now dealing with fever patients in addition to providing general patient care, which adds to their workload. Although we are a private practice that can usually perform esophagogastroduodenoscopy (EGD), we need to be more careful to prevent infection during the procedure. Additionally, although recent data suggest that the risk is low, it has been reported that endoscopists are at risk for COVID‐19 virus infection during endoscopy.^2^, ^3^ As the EGD is carried out by a small number of staff in a private clinic, a safe environment is required for both staff and patients, as well as high efficiency. Therefore, since January 2021, our clinic introduced Endo barrier as an infection protection system for all patients during the EGD. Endo barrier can be effective in preventing the spread of droplets and aerosols generated by patients undergoing EGD by using continuous suction.^4^, ^5^

However, because patients enter a space covered with vinyl film, there were initial concerns that they would complain of pressure and breathlessness. Concerns have been raised about the system's potential longer preparation time and increased workload for staff. The burden on patients and medical staff has not been examined previously, despite reports on devices to prevent COVID‐19 infection during EGD. Therefore, we investigated patient discomfort and satisfaction with the use of the Endo barrier to prevent COVID‐19 infection during EGD. Furthermore, to assess the degree of burden on the medical staff, the preparation time was measured, and its efficiency was evaluated.

## MATERIALS AND METHODS

### Endo barrier

**FIGURE 1:**
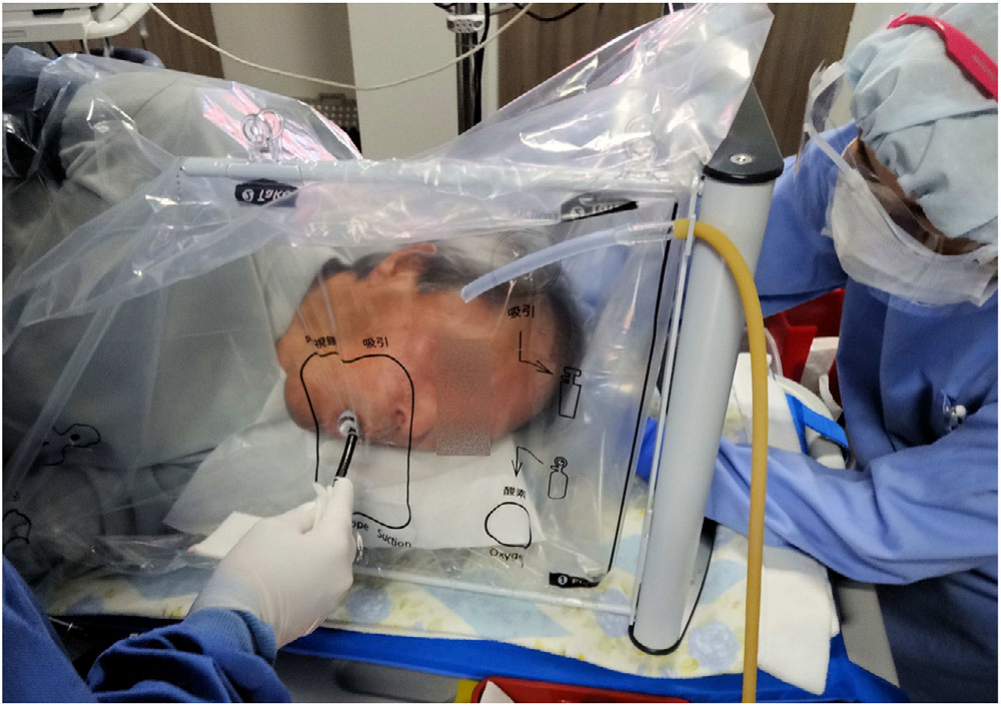
EGD with the Endo barrier. The endoscopist and medical staff perform the EGD with PPE using the Endo barrier. A scope insertion port and a suction insertion port are created in the thin plastic film

Endo barrier is a new infection prevention system against viruses and bacteria, including severe acute respiratory syndrome coronavirus 2 (SARS‐CoV‐2), during EGD. A metal‐framed box is placed over the patient's torso and covered with a thin vinyl film. The vinyl film is disposable and can be attached and removed for each case. Additionally, the suction tube can be placed on the head side to create negative pressure and prevent the spread of droplets, as well as to prevent an increase in CO_2_ concentration in the patient's space. The endoscope can be introduced by creating a tiny hole in the thin vinyl film with a finger as it includes minute perforated lines that are easily punctured (Figure 1).^4^, ^5^ At the end of the procedure, after the patient's cough reflex has subsided, the suction tube that was placed inside the Endo barrier is cut and discarded along with the vinyl film to prevent the spread of aerosols. After that, the endoscopy room doors are opened for each case, and sufficient ventilation is provided to prevent infection, including that of the medical staff.

### Subjects

Patients with clinical evidence of severe diseases, such as an American Society of Anesthesiologists classification of 4 or 5 at the time of EGD, and patients who refused to utilize the Endo barrier due to significant anxiety or other reasons were excluded from this study. In reality, one case (a middle‐aged woman) complained of reluctance to have her upper body in a space surrounded by vinyl film however she was ultimately able to undergo the procedure after receiving careful explanations. Fortunately, all patients during the study period were able to participate and answer questions. A private clinic performed 788 consecutive cases (445 females and 343 males; mean age, 63.7 years) of EGD using Endo barrier from January 2021 to February 2022. Note that, 549 patients were carried out EGD with conscious sedation (Table 1). A questionnaire was used to survey patients after the procedure on four points: discomfort (feeling of pressure, breathlessness) and good points (feeling of relief, satisfaction) using the visual analog scale (VAS). Additionally, the staff's preparation time for the Endo barrier was measured.

**TABLE 1:** Characteristics of all patients

Number of patients	788
Mean Age,	63.7 ± 13.5
Gender, n (%)	
Female	445 (56.5)
Male	343 (43.5)
ASA classification, n (%)	
ASA 1	206 (26.1)
ASA 2	518 (65.7)
ASA 3	64 (8.1)
The total number of EGD in life, n (%)	
First	114 (14.5)
Several times	674 (85.5)
Sedation, n (%)	
With sedation	549 (69.7)
Without sedation	239 (30.3)

## METHODS

### Examination 1

Survey method: After the EGD procedure, patients were given a questionnaire to evaluate the Endo barrier using VAS for four items. The endoscopist explained the study and the questions to all patients before the EGD, and the medical staff gave the questionnaire to the patient to fill out after the procedure. A score of 0 for “feeling of pressure” was defined as no pressure at all, and 10 as too much pressure. For “breathlessness”, a score of 0 was no breathlessness at all, and 10 was very breathless. For “feeling of relief”, a score of 0 indicated no relief at all, while 10 was very relieved. For “satisfaction”, a score of 0 was considered not at all unsatisfactory, and 10 was considered very satisfactory. In addition, all patients were divided into two groups according to sedation status: with sedation (549, 69.7%) and without sedation (239, 30.3%), and their scores were compared (Table 3).

### Examination 2

Preparation time measurement: The time to place the vinyl film in the metal box was defined as the Endo barrier preparation time and was measured for each patient by a different staff member using a stopwatch. The preparation times before the 22nd case were excluded because the staff was unfamiliar with the setup. The first few cases in particular took a long time to set up, and no actual time measurements could be made.

Statistical analysis: Data are expressed as mean ± SD. Statistical comparisons of the data from patients in the two groups were performed using the Mann–Whitney *U* test for numerical data with R statistical software (ver. 4.0.4). The statistical significance of differences was determined using a *p*‐value of less than 0.05.

Ethics: The present study was approved by the Ethics Committee of the Kagawa Medical Association (2020‐04) on February 5th, 2021

## RESULTS

The scores for “feeling of pressure” and “breathlessness” felt during the examination were 0.38 ± 1.37 and 0.25 ± 1.14, respectively. Overall, the scores for pressure and breathlessness were low. In contrast, the patients' evaluations for “feeling of relief” and “satisfaction”, were generally favorable, with scores of 8.53 ± 2.38 (10: reassuring) and 8.50 ± 2.39 (10: very satisfied), respectively (Table 2).

**TABLE 2:** Result of Examination 1

Visual Analogue Scale	
Feeling of pressure	0.38 ± 1.37
Breathlessness	0.25 ± 1.14
Feeling of relief	8.53 ± 2.38
Satisfaction	8.50 ± 2.39

The results of the questionnaire using the visual analog scale (VAS) indicate that the Endo barrier was a safe device for patients, with little pressure or breathlessness.

Comparing the scores of the sedated and non‐sedated groups, both “feeling of pressure” and “breathlessness” were significantly lower in the sedated group (*p* < 0.0001). In contrast, although there was no significant difference in “feeling of relief”, the sedation group had a higher value, and “satisfaction” had a significantly higher value (*p* < 0.01), indicating that the overall results were better in the sedation group (Table 3).

**TABLE 3:** Result of VAS between patients with and without sedation

	With sedation	Without sedation	*P*‐value
Number of patients	549	239	
Feeling of pressure	0.25 ± 1.08	0.71 ± 1.83	< 0.0001
Breathlessness	0.17 ± 0.95	0.44 ± 1.48	< 0.0001
Feeling of relief	8.63 ± 2.32	8.31 ± 2.49	0.05
Satisfaction	8.64 ± 2.30	8.17 ± 2.58	< 0.01

Abbreviation: VAS, visual analogue scale.

Although the overall results were better in the sedation group, the overall evaluation of the non‐sedated group was also favorable.

**FIGURE 2:**
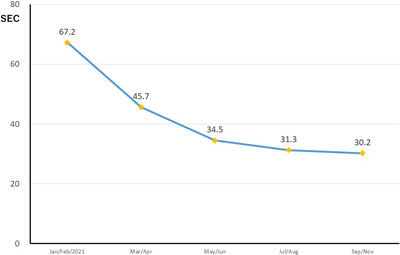
Result of Examination 2. Changes in end‐barrier preparation time. The preparation time was reduced from about 67 s in the first 2 months after introduction to about 30 s after 4 months.

Although it took about 67 s to prepare the system in the first 2 months, it became possible to do so in about 30 s from the fifth month after introduction, and the burden on staff prior to the EGD procedure was almost eliminated (Figure 2 and Video 1).

**VIDEO 1:** The Endo barrier^®^ preparation. This video shows the preparation of the Endo barrier^®^ at sixmonths after introduction, and the system can be prepared smoothly and quickly.

## DISCUSSION

This study investigated patient discomfort and satisfaction during the use of the Endo barrier and further assessed the burden on medical staff by measuring system preparation time. The results indicated that the Endo barrier, a device for the prevention of COVID‐19 infection used during EGD, is satisfactory for both patients and medical staff. No prior study had looked at the effects on patients and medical staff, despite reports on devices to prevent COVID‐19 infection during EGD.

The possibility of COVID‐19 being transmitted from human to human via endoscopy has already been reported and is indicated in World Endoscopy Organization guidelines.^6^ In accordance with these, our clinic verifies in advance via a questionnaire that the patient has no upper respiratory tract infection symptoms or contact with infected persons within 2 weeks. Additionally, all endoscopists and medical staff who participate in the EGD procedure wear appropriate personal protective equipment (PPE), such as waterproof disposable gowns, surgical masks, gloves, face guards, and caps.^7^ Although the risk of infection to endoscopists and medical staff during the procedure is reported to be minimal when PPE is used appropriately,^2^, ^3^ the risk of exposure to SARS‐CoV‐2 is still perceived to be higher than in a routine medical consultation. Mouthpieces or surgical masks with droplet protection and devices to enclose patients in shield barriers have been invented and reported as further infection control methods, but their efficacy has not yet been thoroughly studied, and they are difficult to prepare.^8^, ^9^, ^10^, ^11^, ^12^, ^13^, ^14^, ^15^

The cost of a surgical mask with a hole is estimated to be 0.5$ per case, which is inexpensive, but in a pilot study, it was reported that some contamination was observed in the PPE of the endoscopist, although no contamination was observed in the surrounding medical staff. ^9^ A three‐dimensional printer face mask with plastic frame blocks most microdroplets to the surrounding area, and the cost is estimated to be 1$, but these are not yet commercially available.^12^ A prototype box type costs 40$ and is effective for contamination, but its effectiveness for the prevention of fine aerosols has not been proven.^13^ Other devices cost about 1$, but their effectiveness in preventing aerosol diffusion has not been proven with evidence.

Endo barrier, which became ready for introduction in January 2021, was adopted by our facility because of its advantages over competing products. One advantage is that the thin plastic film has minute‐perforated lines that can be easily pierced, enabling easy endoscope insertion. In essence, this means that an insertion route can be easily and quickly created when necessary to insert not only an endoscope but also a suction tube for CO_2_ and aerosol aspiration or a tube for oxygen administration. By adopting that specification, the possibility of an elevated CO_2_ concentration and the requirement for decreased oxygen saturation treatments have been eliminated because the patient is positioned in a half‐closed space. To verify the effectiveness of the End barrier, a pilot study using smoke to verify that there is no leakage of smoke and visualized the degree of the particle (simulated aerosols) and food coloring (simulated droplets) scattering has shown that the End barrier is effective in reducing direct virus exposure.^5^ Fortunately, because of the above measures, no patient, staff, or endoscopist has ever been infected with COVID‐19 due to EGD at our institution.

In contrast, its disadvantages were that the patient enters an enclosed space and may feel pressured and breathless as well as the time required to prepare the system. In this study, patients did not feel much stress or breathlessness during the procedure, which is a welcome result. However, the patients who enrolled in this study were informed in advance about the necessity of EGD using the Endo barrier, and their consent was obtained, resulting in less pressure and breathlessness. A comparison of the sedation and non‐sedation groups showed that the sedation group performed better in all result scores. Sedatives have the effect of reducing anxiety, and the “feeling of pressure” and “breathlessness” were naturally favorable with conscious sedation. “Satisfaction” was also better in the sedation group, suggesting that sedation is better for patients in the EGD procedure under highly anxious conditions. Conversely, the overall evaluation of the non‐sedated group was also favorable, and we are convinced that the use of the Endo barrier provides a satisfactory and comforting endoscopic procedure. The results were satisfactory because the patients felt that the device was useful in preventing COVID‐19 infection, and we were able to perform the EGD with a high degree of satisfaction. The inability of endoscopists to view the patient's facial expressions throughout the process, the difficulty of performing a precise scope manipulation, and the system's large space requirement were additional problems. As we became accustomed to using it, the above disadvantages were resolved naturally.

Immediately after the introduction, a preparation time of more than 60 s was needed, but eventually, it became possible to reduce this, enabling an efficient procedure. In the end, there was no disruption to the routine EGD process and no burden on the medical staff.

By using the Endo barrier, the new findings showed that not only patients but also medical staff had a safe and comfortable procedure even during the COVID‐19 pandemic. For these reasons, the Endo barrier is now an indispensable device because it eliminates concerns about the prevention of COVID‐19 infection in both patients and staff.

This study had several limitations. The observation study included a small number of cases, was not blind and was conducted at a single center. There was insufficient evidence that the Endo barrier prevented COVID‐19 infection, although this is also true of other COVID‐19 infection prevention devices used during EGD. It should be noted that the system cost was 1.5$ per person. A future large prospective study is anticipated to be carried out as we think the Endo barrier has the potential to become a useful tool for preventing COVID‐19 infection during EGD. Furthermore, in the future even in the COVID‐19 pandemic, it may no longer be necessary to prepare and replace PPE for each patient.

In conclusion, the Endo barrier is an easy‐to‐implement tool to prevent COVID‐19 infection in private clinics, and both patients and staff are highly satisfied with the device.

## CONFLICT OF INTEREST

None.

## References

[1] World Health Organization (WHO), Others . COVID‐19 weekly epidemiological update *[Internet]*. Geneva, Switzerland: WHO; [cited 2022 Sep 28]. Available from: https://www.who.int/publications/m/item/weekly‐epidemiological‐update‐on‐covid‐19—28‐september‐2022

[2] Repici A , Aragona G , Cengia G *et al*. Low risk of COVID‐19 transmission in GI endoscopy. Gut 2020; 69: 1925–7. 32321857 10.1136/gutjnl-2020-321341

[3] Niikura R , Fujishiro M , Nakai Y *et al*. International observational survey of the effectiveness of personal protective equipment during endoscopic procedures performed in patients with COVID‐19. Digestion 2021; 102: 845–53. 33592610 10.1159/000513714 PMC8018186

[4] Fujihara S , Kobara H , Nishiyama N *et al*. Clinical efficacy of novel patient‐covering negative‐pressure box for shielding virus transmission during esophagogastroduodenoscopy: A prospective observational study. Diagnostics 2021; 11: 1679. 34574020 10.3390/diagnostics11091679 PMC8470820

[5] Kobara H , Nishiyama N , Oba H *et al*. Verification of negative pressure box for preventing severe acute respiratory syndrome coronavirus 2 (SARS‐CoV‐2) transmission during upper gastrointestinal endoscopy. JGH Open 2021; 5: 825–6. 34263079 10.1002/jgh3.12595 PMC8264236

[6] Guda NM , Emura F , Reddy DN *et al*. Recommendations for the operation of endoscopy centers in the setting of the COVID‐19 pandemic – World Endoscopy Organization guidance document. Dig Endosc 2020; 32: 844–50. 32569438 10.1111/den.13777 PMC7361408

[7] Chiu PWY , Ng SC , Inoue H *et al*. Practice of endoscopy during COVID‐19 pandemic: Position statements of the Asian Pacific Society for Digestive Endoscopy (APSDE‐COVID statements). Gut 2020; 69: 991–6. 32241897 10.1136/gutjnl-2020-321185 PMC7211066

[8] Bojórquez A , Larequi FJZ , Betés MT *et al*. Commercially available endoscopy facemasks to prevent aerosolizing spread of droplets during COVID‐19 outbreak. Endosc Int Open 2020; 8: E815–6. 32529025 10.1055/a-1180-8355 PMC7280021

[9] Maruyama H , Higashimori A , Yamamoto K *et al*. Coronavirus disease outbreak: A simple infection prevention measure using a surgical mask during endoscopy. Endoscopy 2020; 52: 461–2. 10.1055/a-1220-6024 PMC7724578 32818997

[10] Endo H , Koike T , Masamune A . Novel device for preventing diffusion of aerosol droplets from subjects undergoing esophagogastroduodenoscopy during COVID‐19 pandemic. Dig Endosc 2020; 32: e140–1. 32696996 10.1111/den.13772 PMC7405108

[11] Lazaridis N , Skamnelos A , Murino A *et al*. Double‐surgical‐mask‐with‐slit” method: Reducing exposure to aerosol generation at upper gastrointestinal endoscopy during the COVID‐19 pandemic. Endoscopy 2020; 52: 928–9. 32967022 10.1055/a-1198-5471 PMC7516391

[12] Maehata, T , Yasuda H , Kiyokawa H *et al*. A novel mask to prevent aerosolized droplet dispersion in endoscopic procedures during the coronavirus disease pandemic. Medicine 2021; 100: e26048. 34190142 10.1097/MD.0000000000026048 PMC8257832

[13] Sagami R , Nishikiori H , Sato T *et al*. Endoscopic shield: Barrier enclosure during the endoscopy to prevent aerosol droplets during the COVID‐19 pandemic. Video GIE 2020; 5: 445–8. 32395674 10.1016/j.vgie.2020.05.002 PMC7211574

[14] Kobara H , Nishiyama N , Masaki T . Shielding for patients using a single‐use vinyl‐box under continuous aerosol suction to minimize SARS‐CoV‐2 transmission during emergency endoscopy. Dig Endosc 2020; 32: e114–5. 32492223 10.1111/den.13713 PMC7300621

[15] Kikuchi D , Suzuki Y , Nomura K *et al*. New safety measure for the endoscopic procedures during the COVID‐19 pandemic: New STEP. Video GIE 2020; 5: 634–6. 32959026 10.1016/j.vgie.2020.07.014 PMC7495179

